# Influence of students’ personality, gender, income and age on their intentions to create new information technology and telecommunications ventures

**DOI:** 10.1371/journal.pone.0284488

**Published:** 2023-07-21

**Authors:** Gustavo Barrera-Verdugo, Jaime Cadena-Echverría, Antonio Villarroel-Villarroel, Michelle Contreras-Fuenzalida

**Affiliations:** 1 Faculty of Engineering and Business, Universidad de Las Américas, Providencia, Santiago, Chile; 2 Facultad de Ciencias Administrativas, Escuela Politécnica Nacional, Quito, Ecuador; University of Kurdistan Hewler, IRAQ

## Abstract

Businesses associated with information technology and telecommunications have increased in importance since the start of the COVID-19 pandemic due to transformations in working and buying. Currently, universities in Latin America are making efforts to strengthen entrepreneurial attitudes and skills in their students. In this context, it is of interest to understand how the combination of personality traits, gender, income and age/generation affect entrepreneurial intentions toward these kinds of businesses. This research analyzes the responses of 788 university students from Chile and Ecuador to an online self-report survey using regression models with the aim of evaluating the effect of Big Five personality traits on the intention to undertake information technology and telecommunications businesses and measuring the moderation of gender, family income, and belonging to the centennial generation. The findings support a significant influence of the traits of neuroticism, openness and conscientiousness on entrepreneurial intention and suggest that female gender and higher family income have a negative impact on the intention to undertake these businesses. Moreover, an important moderating effect of female gender and lower family income levels is supported. This study sheds new light on students’ characteristics that affect their participation in information technology and telecommunications ventures through the identification of a unique combination of relevant personality traits, gender and income levels. The findings are useful for designing and developing appropriate programs aimed at developing this kind of technology business in Latin America as well as promoting the entrepreneurship of groups that express a lower intention to undertake businesses, such as female students.

## Introduction

The pandemic caused by coronavirus disease 2019 (COVID-19) impacted not only people’s health but also people’s lifestyles and the world economy. Many governments were forced to make extreme decisions related to quarantines, causing a slowdown in economic activities and affecting the development of businesses. Faced with this new scenario, entrepreneurs have adapted their activities to face challenges such as digitalization and a lack of financing and human resources. Moreover, they have found new business opportunities associated with technological ventures [1]. In particular, businesses related to information technology and telecommunications services have become increasingly important after the pandemic [2] due to increased requirements for connectivity and remote processes for work, shopping and studies, among other postpandemic activities.

In this new scenario, it is crucial to understand the positive and negative effects of personal characteristics in the development of information technology and telecommunications enterprises. An important personal characteristic that affects business creation is the intention to undertake, defined as a mental state or predisposition that leads an individual to choose self-employment instead of working for other people [3]. Consequently, the study of the personal conditions that affect the intention to undertake is a line of research that is currently of great interest. These determinants can be divided into two groups: first, a group of psychological variables, such as motivation and self-efficacy; second, a group of demographic conditions, such as gender, age and family income. Among the psychological variables that affect entrepreneurial intention, the literature has highlighted the influence of personality traits [4]. Personality is defined as individual differences in characteristic patterns of thinking, feeling, and behaving that distinguish individuals [5]. Various investigations have supported the effect of personality traits on the intention to undertake in business areas such as tourism [6], social entrepreneurship [7] and agriculture [8].

To date, the effect of the personality traits of Latin American students on their intention to start a business in the information technology and telecommunications area has not been analyzed in depth. Studies in the region tend to analyze the effect of students’ demographic characteristics or the effects of entrepreneurial education on students’ entrepreneurial intentions [9–11] without examining the effect of personality on the intention to start specific new ventures, such as computer and telecommunications businesses. This lack of knowledge is relevant because this business area has increased during the last two years after the start of the COVID-19 pandemic due to growing demands for remote work, videoconference communication, telemedicine and online shopping. Similarly, the moderation of students’ gender, age and family income level on the relationship between personality traits and entrepreneurial intentions has not been extensively evaluated in this region. Although some research has explored the influence of gender and income on the intention to start a business in Latin America [12,13], to date, the study of the moderating effect of these variables on the relationship between personality traits and entrepreneurial intention in this region is limited. The analysis of this moderation is useful to identify profiles of students with a greater predisposition to undertake this type of business since the importance of gender, age and income level in understanding entrepreneurial behaviors has been widely supported [14–17]. This research seeks to address this knowledge gap. Therefore, the aim of the study is to evaluate the influence of personality traits on Latin American university students´ intention to undertake information technology and telecommunications businesses by analyzing the moderating role of the aforementioned demographic variables. Obtaining a better understanding of the personal characteristics that affect entrepreneurial intention in information technology and telecommunications businesses among Latin American university students is crucial since there are deficiencies in the availability of programmers and the supply of software in the region. Additionally, there is a significant gap in female participation in science, technology, engineering and mathematics (STEM) subjects in countries in this region [18]. The results of this study can guide the development of programs and projects to strengthen students’ entrepreneurial intentions in Latin America, which is particularly important due to the worrying deficit of technological talent in these countries [19].

## Theoretical frame

### Personality

Personality is defined as a person’s dynamic organization of psychophysical systems, which involves patterns of behaviors, thoughts and feelings that identify the individual [20]. This concept can be understood as individuals’ consistent responses to external stimuli [21]. Therefore, personality refers to individual differences in characteristic patterns of thinking, feeling, and behaving that distinguish people [5]. Engler [21] held the view that personality is dynamic (moves and changes), organized (structured), psychophysical (involving both mind and body), determined by the past and predisposed to the future (structured), and characteristic (unique to each individual).

An important referential framework is trait theory, which states that personality is constituted through a unique configuration of traits and that these traits are defined as individuals’ dispositions that allow their behaviors to be recognized and differentiated [22]. A model based on personality traits with high application in the last 30 years is the Big Five, which was developed by McCrae and Costa [23]. This referential framework states that there are five major factors (traits) that configure the individual personality through their combination. The Big Five model of personality has become an open, powerful and comprehensive model for analyzing the traits that people use to describe themselves and others [24]. According to McCrae and Costa [23] and John et al. [25], the traits of the Big Five model of personality are as follows: 1- extraversion, which is related to positive emotions, sociability, cheerful, energetic and assertive behaviors, and participation in social activities; 2- agreeableness, which represents trusting, helpful and selfless thoughts, feelings and behavior as well as a prosocial and community orientation; 3- openness to experience, which implies being creative, imaginative, curious and flexible in thinking, feeling and behaving; 4- conscientiousness, which is related to being organized, reliable, punctual, hardworking, determined and self-disciplined; and 5- neuroticism, which is associated with the manifestation of negative emotions such as anxiety or anger and with negative thoughts and impulsive behaviors.

Research during the last two decades has supported the influence of personality on the expression of entrepreneurial attitudes and behaviors. In this line, López-Núñez et al. [26] demonstrated that it is possible to identify an entrepreneurial profile based on personality traits. In particular, personality differences between entrepreneurs and nonentrepreneurs have been recognized [27], the personality traits of managers who develop innovative business models have been investigated [28], and the influence of personality traits on the success of ventures [29] and on venture internationalization [30] have been analyzed.

Among the variables studied in the field of entrepreneurial behavior, entrepreneurial intention is widely valued. This concept can be defined as a state of mind that leads an individual to choose self-employment as opposed to working for others [3]. It is important because it is a crucial stage of the entrepreneurial process that influences subsequent entrepreneurial behaviors [14]. With respect to personality traits, several investigations have shown that personality is a predictor of individual entrepreneurial intentions [31]. Sahinidis et al. [32] demonstrated that the traits of openness to experience, conscientiousness and extraversion positively influence entrepreneurial intention, while agreeableness, neuroticism and risk aversion show an opposite effect. Khan et al. [33] argued that extraversion and agreeableness are the most influential traits for entrepreneurial intention in sustainable businesses in Pakistan. Hossain et al. [6] revealed that agreeableness, conscientiousness, extraversion, emotional stability, openness, self-efficacy and social support significantly influence social entrepreneurial intention. Mei et al. [34] showed that emotional stability (minus neuroticism), conscientiousness and extraversion are positively associated with entrepreneurial intention. Awwad and Al-Aseer [35] argued that conscientiousness and openness are associated with entrepreneurial intention, and Ahmed et al. [36] found that conscientiousness has a significant positive influence on entrepreneurial intention, while the traits of extroversion, openness to experience, neuroticism and agreeableness do not.

The aforementioned studies were conducted in contexts other than Latin America, and they did not focus on evaluating the influence of personality on entrepreneurial intention in emerging business areas due to the social and economic changes generated by the COVID-19 pandemic, such as information technology and telecommunications businesses. Considering the evidence that supports the different effects of personality on entrepreneurial intention in different geographic zones and/or business areas, this research proposes that the personality of Latin American university students should influence their intention to undertake information technology and telecommunications businesses through a unique configuration of personality traits. Thus, the following hypothesis is proposed:

- Hypothesis 1: Latin American university students’ personality traits influence their intention to undertake information technology and telecommunication businesses.

### Demography

Demography is defined as the scientific study of the human population with respect to size, structure and composition [37]. Demography bases its analysis on variables such as age, sex, income levels, occupation, and marital status. Research has shown that demographic characteristics play an important role in predicting entrepreneurial intention [38,39]. Recently, Kar and Ahmed [40] examined the relationship between demographic conditions and the intention to abandon a venture by studying young entrepreneurs aged 18 to 29 years old in Ethiopia. Their results suggest that gender, previous work experience and family occupation have a statistically significant effect.

A demographic variable that has generated high interest in recent decades is gender, and several authors have argued for differences in entrepreneurial intention between men and women [14,15,41,42]. Most previously published evidence supports higher entrepreneurial intention among men [43]. Moreover, gender has been incorporated as a moderating variable in the study of entrepreneurial intention. For example, Hassan et al. [44] demonstrated that gender negatively moderates the relationship between opportunity recognition intention and entrepreneurial self-efficacy, Moreno-Gomez et al. [45] found that gender moderates the relationship between parental role models and entrepreneurial intention, and Elnadi et al. [46] argued that female gender negatively moderates the relationship between self-efficacy and entrepreneurial intention. Therefore, considering the evidence that supports lower entrepreneurial intention among women and the moderating effect of gender, this research proposes that the influence of Latin American university students’ personality on their intention to start information technology and telecommunication businesses should be negatively moderated by female gender. Consequently, the following research hypothesis is proposed.

- Hypothesis 2: Female gender negatively moderates the influence of Latin American university students’ personality traits on their intention to undertake information technology and telecommunications businesses.

In a complementary way, several studies have identified a relationship between income, or socioeconomic level, and entrepreneurial intentions. In this regard, it is possible to identify two lines of evidence. First, some research has argued that people with high incomes who have support networks and more access to financing demonstrate higher entrepreneurial intention [16,38,47,48]. A second line of evidence posits that poverty associated with low income promotes greater entrepreneurial intention [49,50]. This view holds that business creation is related to income needs; that is, for poor people, creating their own ventures is not an aspiration but rather it is a necessity since low family income pushes them toward business creation [51]. In summary, previous research tends to attribute opportunity-based entrepreneurship to middle-income and high-income individuals and to link poverty with necessity-based entrepreneurship [52,53].

This research evaluates the entrepreneurial intention of university students with medium and low family incomes who study in universities in Latin American countries that face important social and economic problems that have intensified with the COVID-19 pandemic. Therefore, this research proposes that belonging to the first quintile of family income (lower income) positively moderates the relationship between personality traits and entrepreneurial intention toward information technology and telecommunication businesses since it should boost entrepreneurial intention for necessity, understanding that entrepreneurship for necessity is the choice to start a new business because other options for work are not available or unsatisfactory [54]. Consequently, the following hypothesis is proposed.

- Hypothesis 3: Belonging to the first income quintile positively moderates the influence of Latin American university students’ personality traits on their intention to undertake information technology and telecommunications businesses.

Additionally, several studies have analyzed changes in entrepreneurial intention across the course of life. Zhang et al. (2018) [55] posited that the intention to undertake a full-time business begins to decline after the age of 30. Gielnik et al. (2018) [56] stated that younger people have a longer future time perspective than older people; therefore, younger people find more opportunities and then tend to exploit these opportunities through new venture creation. Kautonen et al. [57] argued that people over the age of 50 who have spent most of their lives working in industrial jobs have less entrepreneurial intention and less social support to start a business. Hatak et al. [58] revealed that elderly employees are less willing to be entrepreneurs and that their entrepreneurial intention is lower the more they identify with their job. Recently, Maalaoui et al. [17] showed that people older than 45 years with higher entrepreneurial intentions tend to feel rejuvenated; hence, it is necessary to stimulate entrepreneurial potential in these age groups.

In relation to the propensity of the centennial generation–also called Generation Z–to participate in technological businesses, research evidence has shown that centennials value new technologies in fields such as shopping, social relationships and work [59–61]. It has also been suggested that centennials tend to value new ventures based on new technology [62], which is consistent with their status as digital natives, i.e., they are a hypercognitive generation that can interact with those equal in age through social media sites [63]. Complementarily, age has been incorporated as a moderating variable in studies of entrepreneurial intention [64]. In this sense, Liao et al. [65] showed that age significantly moderates the relationship between cognitive background and entrepreneurial intention. Based on these previous research results that suggest that younger age positively affects the recognition of entrepreneurial opportunities and supports centennials’ greater valuation of new technologies, this research proposes the following hypothesis:

- Hypothesis 4: Belonging to the centennial generation positively moderates the influence of Latin American university students’ personality traits on their intention to undertake information technology and telecommunications businesses.

## Methodology and methods

### Measurement

The research was quantitative and used self-administered online questionnaires, which were distributed through the SurveyMonkey platform. The Midlife Development Inventory (MIDI) personality scales by Lachman and Weaver [66] were used to measure personality traits according to the Big Five model; this scale has been widely recognized and applied in recent studies [67–69]. The MIDI scale measures the Big Five personality traits, agreeableness, neuroticism, openness, conscientiousness, and extraversion, through the recognition of individual differences assessed with four levels representing the intensity of each personal trait. These four levels of measurement defined by Lachman and Weaver [66] are as follows: not at all (1), a little (2), some (3), and a lot (4). The question in MIDI scale is “Please indicate how well each of the following describes you”. After obtaining the responses to the online survey, the scores for each personality trait were calculated using the procedure defined by Lachman and Weaver [66], which is based on an arithmetic mean of the items related to each trait. Table 1 describes the qualities associated with agreeableness, neuroticism, openness, conscientiousness, and extraversion in MIDI scale.

**Table 1:** Personality trait assessment.

.Personality Trait	Characteristics	Scale
**Agreeableness**	Caring, helpful, soft-hearted, warm, sympathetic.	Not at all (1) A little (2) Some (3) A lot (4)
**Neuroticism**	Moody, worrying, nervous, calm (reverse).	
**Openness**	Imaginative, creative, broadminded, intelligent, curious, sophisticated, adventurous.	
**Conscientiousness**	Organized, responsible, hardworking, careless (reverse).	
**Extraversion**	Talkative, outgoing, friendly, lively.	

Note: Characteristics and measurement levels defined in the Midlife Development Inventory (MIDI).

Entrepreneurial intention was assessed using the scale created by Liñán and Chen [70], which has also been validated and used in recent studies [71–73]. The scale of Liñán and Chen [70] was adapted to evaluate the entrepreneurial intention to create businesses in the information technology and telecommunications sector. Consequently, their 6 statements were adapted as follows: 1- I am ready to do anything to be an entrepreneur in the information technology and telecommunications industry; 2- my professional goal is to become an entrepreneur in the information technology and telecommunications industry; 3- I will make every effort to start and run my own firm in information technology and telecommunications industry; 4- I am determined to create a firm in the future in the information technology and telecommunications industry; 5- I have seriously thought about starting a company in the information technology and telecommunications industry; 6- I have the firm intention to start a firm in the information technology and telecommunications industry someday. All items were evaluated with a seven-point Likert scale ranging from "strongly disagree" (1) to "strongly agree" (7). The seven-point scale was used because it included more options than the five-point scale, allowing for the observation of more levels [74].

Personality scale and entrepreneurial intention scale items were translated from English to Spanish to collect responses from Spanish-speaking students and then translated back into English for presentation in this research. Students born since 1997 were considered to belong to the centennial generation based on the definition by McGorry and McGorry [75]. Belonging to the centennial generation was identified with a dummy variable that coded centennials with the number "1" and older people with the number "0". Belonging to the first income quintile was identified through a dummy variable that coded the first 5 deciles of family income, which is equivalent to the first quintile of family income, with the number "1" and income quintile 2 with the number "0". Gender was also evaluated with a dummy variable that identified the female gender with the number "1" and the male gender with "0".

### Sample

The responses of 788 university students from Chile and Ecuador, most of whom were enrolled in majors associated with engineering and business, were analyzed. The response period was from April 2022 to August 2022. The students were selected through nonprobability convenience sampling, which is frequently used in research that asks university students about entrepreneurial behaviors [35,76,77]. The survey included a written informed consent in accordance with the guidelines of the Ethics Committee of Universidad de Las Américas (ID: 44/2022). Only students who accepted the written informed consent request had access to the online survey questions. Likewise, only correctly completed responses were analyzed.

The sample included 221 (28.05%) students from Chile and 567 (71.95%) students from Ecuador. The respondents were enrolled in programs at Universidad de Las Américas in Chile (221), Escuela Politécnica Nacional in Ecuador (356), Universidad de las Fuerzas Armadas in Ecuador (161) and other universities in Ecuador (52). A total of 95.02% of those evaluated in Chile were in the Metropolitan Region of Santiago, and 95.10% of those evaluated in Ecuador were in the city of Quito. The majority of the students responded that they were enrolled in business, economics or engineering majors, particularly engineering, bachelor’s or technical studies in business administration (247, 31.35%); commercial engineering (83, 10.53%); production engineering (62, 7.87%); bachelor’s degree in marketing or commerce (54, 6.85%); industrial engineering (51, 6.47%); bachelor’s degree in economics (51, 6.47%); engineering in mathematics (42, 5.33%); other engineering areas, such as mechanics, electronics, computer science, chemistry and the environment (149, 18.91%); and other majors, such as auditing and public administration (49, 6.22%).

The gender distribution was 411 (52.16%) male students and 377 (47.84%) female students. The mean age of those evaluated was 24.38 years, and the standard deviation of age was 6.62 years. Likewise, 505 (64.09%) students belonged to the first quintile of family income (lowest 5 income deciles), and 283 (35.91%) belonged to the second quintile (highest 5 income deciles). According to McGorry and McGorry’s [74] classification, 606 (76.90%) students were categorized as belonging to the centennial generation (18 to 25 years old), and 182 (23.10%) students were categorized as older adults (26 or older).

### Statistical analysis

Since this research sought to evaluate the effect of personality traits and demographic variables on the intention to undertake information technology and telecommunications business activities, a linear regression analysis was performed. The entrepreneurial intention to undertake information technology and telecommunications businesses was included as the dependent variable, and the five personality traits, female gender, first income quintile and belonging to the centennial generation, were used as independent variables. As previously stated, a measurement parameter associated with each personality trait was obtained through the procedure of Lachman and Weaver [66]. The arithmetic mean of the six entrepreneurial intention statements of Liñán and Chen [70] was used as the dependent variable in the regression models because the Cronbach’s alpha coefficient of these six statements was greater than 0.9 (0.95), indicating good reliability for the instrument [78], and because the Spearman correlation coefficients for these six items showed values between 0.49 and 0.91 that were significant with 99% confidence.

The overall fit of the linear regressions was tested with the F-Fisher test. The significance of the regression coefficients was evaluated based on the p values. Moreover, the assumptions of the regression models were assessed; specifically, the assumption of linearity was tested for linearity using the correlation coefficient, the assumptions of normality and homoscedasticity of the residuals were checked using frequency histograms of residuals and plots of residuals, and the absence of multicollinearity of the independent variables was assessed using the variance inflation factor. Variables representing the multiplication of female gender, first income quintile and belonging to the centennial generation with statistically significant personality traits in the unmoderated regression model were created to evaluate the moderating effect of these demographic characteristics. The statistical significance of their coefficients was then evaluated using the P>|t| parameter. Prior to the regression analysis, an analysis of central tendency and comparison of frequency distribution by demographic groups was conducted using the arithmetic mean and Wilcoxon-Mann-Whitney test. The Wilcoxon-Mann-Whitney test was performed because the data were not normally distributed [79]. The normal distribution of the variables was examined through the Shapiro‒Wilk test. Statistical analysis was performed with STATA version 16.

## Results

### Central tendency analysis

The analysis in Table 2 compares the arithmetic means of personality traits and the intention to undertake by gender, family income quintile and age generation. The gender comparison showed higher arithmetic means with respect to trait agreeableness, neuroticism, and conscientiousness in the women’s group; in addition, Table 2 shows a lower arithmetic mean of entrepreneurial intention in the women’s group. The Wilcoxon-Mann-Whitney test supported these differences in data distribution by gender with 99% (p<0.01) or 95% (p<0.05) confidence. The results also showed that lower income respondents (quintile 1) obtained lower means for agreeableness, openness, conscientiousness and extraversion, and, in the opposite direction, showed a higher arithmetic mean for entrepreneurial intention. These differences were supported by the Wilcoxon-Mann-Whitney test with 99% (p<0.01) confidence. Finally, the centennial group obtained a lower arithmetic mean for agreeableness and conscientiousness and extraversion and a higher arithmetic mean for entrepreneurial intention. Such differences were also supported by the Wilcoxon-Mann‒Whitney test with 99% (p<0.01), 95% (p<0.05) or 90% (p<0.10) confidence.

**Table 2:** Personality and entrepreneurial intention by gender, generation and income.

	Woman	Man	P Value	Quintile 1	Quintile 2	P Value	Centennial	Older	*P* Value
**Agreeableness**	3.185	3.096	0.009	3.098	3.211	0.002	3.119	3.203	0.025
**Neuroticism**	2.534	2.361	0.000	2.448	2.438	0.991	2.454	2.411	0.319
**Openness**	3.022	2.993	0.376	2.969	3.074	0.001	3.010	2.997	0.991
**Consciousness**	3.168	3.019	0.000	3.049	3.165	0.001	3.034	3.279	0.000
**Extraversion**	2.901	2.912	0.781	2.851	3.006	0.001	2.888	2.969	0.057
**E. Intention**	3.644	3.952	0.007	3.921	3.597	0.002	3.864	3.606	0.048

**Note:** The *p* value was obtained by performing the Wilcoxon-Mann‒Whitney test. N = 788.

### Regression models

Table 3 presents the model without gender, age group and family income moderation. These demographic variables were incorporated into the model to evaluate their direct effect on entrepreneurial intention. The regression model showed a good fit (Prob > F = 0.00). The results indicate that personality trait of conscientiousness (β = 0.306; P<0.05), which is related to responsibility, organization and discipline, has a positive effect on entrepreneurial intention toward information technology and telecommunications entrepreneurship. In addition, the personality trait of openness (β = 0.360; P<0.05), which is related to being creative, imaginative, curious and flexible in thinking, feeling and behaving, has a positive effect on the entrepreneurial intention of students. In contrast, trait neuroticism (β = -0.246; P<0.05), which is associated with negative emotions and thoughts, shows a negative effect on entrepreneurial intention. Moreover, a negative effect of female gender (β = -0.373; P<0.01) and a positive effect of belonging to the first income quintile (β = 0.394; P<0.01) on entrepreneurial intention are supported. Only a positive effect with 90% confidence of belonging to the centennial generation (β = 0.217; P<0.10) is observed. The three variables with higher standardized regression coefficients (positive or negative) are the first quintile of family income (β = 0.125; P<0.01), female gender (β = -0.124; P<0.01) and personality trait openness (β = 0.110; P<0.01).

**Table 3:** Regression model without moderation.

	Unstandardized coefficient β	Standardized coefficient β	Standard Error	t	P>z	[95% Interval Conf.]	
**Agreeableness**	0.227	0.073	0.150	1.520	0.130	-0.067	0.520
**Neuroticism**	-0.246	-0.071	0.124	-1.980	0.048	-0.490	-0.003
**Openness**	0.360	0.110	0.149	2.410	0.016	0.067	0.653
**Consciousness**	0.306	0.102	0.119	2.590	0.010	0.073	0.539
**Extraversion**	-0.096	-0.037	0.128	-0.750	0.456	-0.347	0.156
**Woman**	-0.373	-0.124	0.109	-3.410	0.001	-0.588	-0.159
**Quintile 1**	0.394	0.125	0.114	3.440	0.001	0.169	0.619
**Centennial**	0.217	0.061	0.132	1.650	0.099	-0.041	0.475
**Constant**	1.704		0.577	2.960	0.003	0.572	2.836

**Note:** Entrepreneurial intention toward information technology and telecommunications businesses is the dependent variable. Number of observations = 788, R-squared = 0.073, Adj R-squared = 0.064, Prob > F = 0.000.

Table 4, presented below, examines the female gender, the first income quartile and the centennial generation moderating effects on the relationship between the neuroticism personality trait and entrepreneurial intention toward new information technology and telecommunications ventures. Female gender, the first income quintile and membership in the centennial generation were only included in the multiplication with the personality trait of neuroticism to avoid multicollinearity effects among the variables. Thus, the variance inflation factor was less than 3.3 for all independent variables, representing the absence of multicollinearity between explanatory variables [80]. The results supported a negative moderation of female gender (β = -0.155; P<0.01) and a positive moderation of family income in quintile 1 (β = 0.161; P<0.01). The moderation of centennial generation membership was not statistically supported (β = 0.083; P>0.10). Moreover, the regression model showed a good fit (Prob > F = 0.00).

**Table 4:** Regression model with moderations related to neuroticism.

	Unstandardized coefficient β	Standardized coefficient β	Standard error	t	P>t	[95% Interval Conf.]	
**Agreeableness**	0.237	0.077	0.150	1.580	0.114	-0.057	0.531
**Neuroticism**	-0.335	-0.097	0.138	-2.420	0.016	-0.606	-0.064
**Openness**	0.357	0.109	0.149	2.390	0.017	0.064	0.650
**Consciousness**	0.308	0.103	0.119	2.590	0.010	0.074	0.542
**Extraversion**	-0.097	-0.037	0.128	-0.760	0.447	-0.349	0.154
**Woman x Neuroticism**	-0.155	-0.134	0.044	-3.520	0.000	-0.242	-0.069
**Quintile 1 x Neuroticism**	0.161	0.131	0.046	3.500	0.000	0.071	0.252
**Centennial x Neuroticism**	0.083	0.061	0.054	1.550	0.121	-0.022	0.189
**Constant**	1.914		0.565	3.390	0.001	0.804	3.024

**Note:** Entrepreneurial intention toward information technology and telecommunications businesses is the dependent variable. Number of observations = 788, R-squared = 0.073, Adj R-squared = 0.064, Prob > F = 0.000.

**Fig 1:**
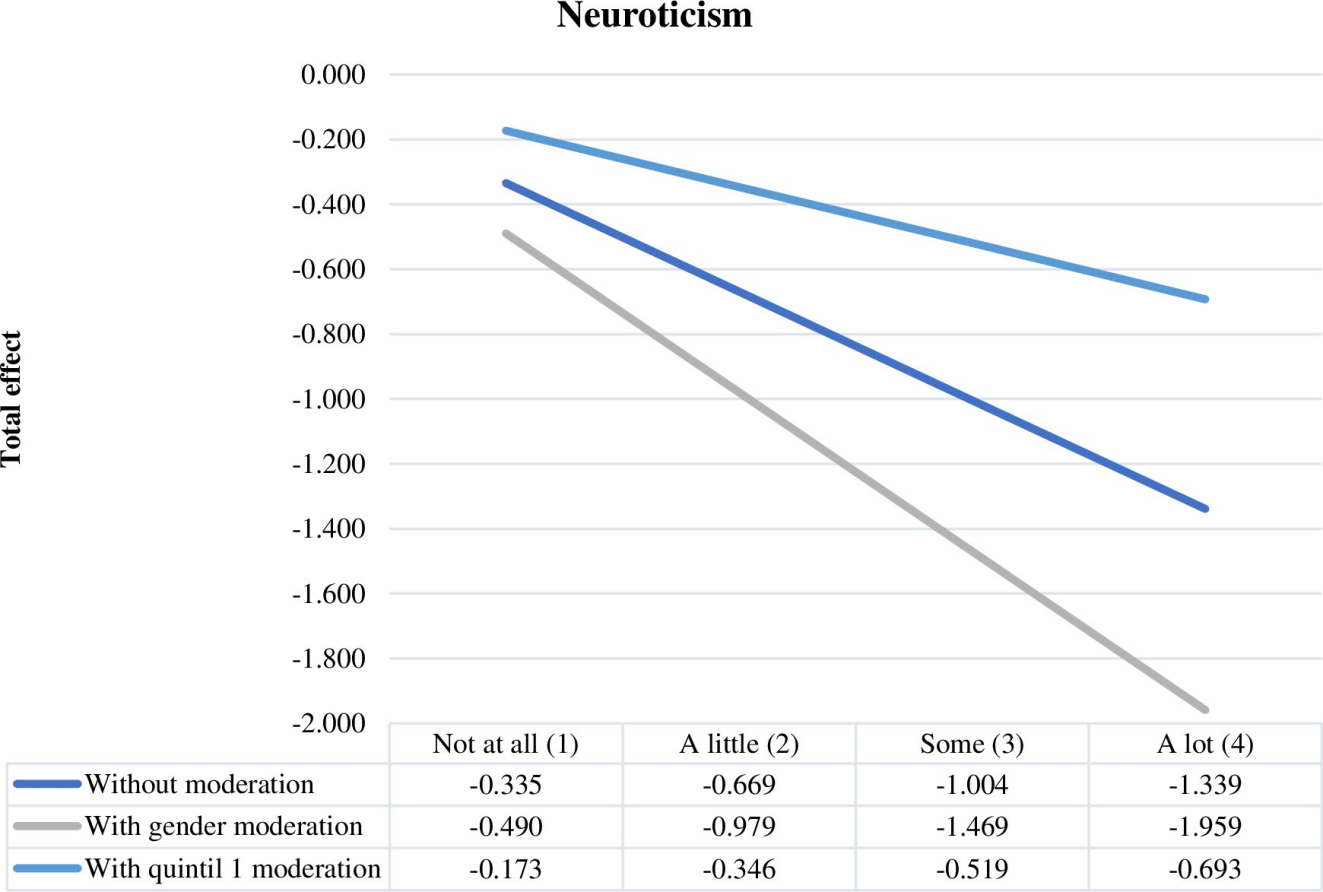
Total effect of neuroticism.

Fig 1 shows the total effect of personality trait neuroticism on the intention to undertake information technology and telecommunications businesses, describing the moderating effect of gender and family income level. Female gender increases the total effect of trait neuroticism, and belonging to the first income quintile decreases this effect.

Table 5 presents the results of the moderation analyses examining the moderating effect of female gender, the first family income quartile and belonging to the centennial generation on the interaction between the openness personality trait and entrepreneurial intention toward new information technology and telecommunications ventures. As in the previous regression model, the negative moderation of female gender (β = -0.117; P<0.01) and the positive moderation effect of lower family income (β = 0.124; P<0.01) were supported. The moderating effect of belonging to the centennial generation was not supported (β = 0.060; P>0.10). Furthermore, the regression model showed a good fit (Prob > F = 0.00).

**Table 5:** Regression model with moderation related to openness.

	Unstandardized coefficient β		Standardized coefficient β	Standard error	t	P>t	[95% Interval Conf.]	
**Agreeableness**		0.224	0.072	0.150	1.490	0.136	-0.071	0.518
**Neuroticism**		-0.249	-0.072	0.124	-2.000	0.045	-0.493	-0.005
**Openness**		0.301	0.092	0.157	1.920	0.056	-0.007	0.608
**Consciousness**		0.294	0.098	0.119	2.470	0.014	0.060	0.528
**Extraversion**		-0.098	-0.037	0.128	-0.760	0.446	-0.350	0.154
**Woman x Openness**		-0.117	-0.120	0.036	-3.260	0.001	-0.187	-0.046
**Quintile 1 x Openness**		0.124	0.121	0.037	3.350	0.001	0.051	0.197
**Centennial x Openness**		0.060	0.053	0.043	1.390	0.166	-0.025	0.145
**Constant**		1.979		0.565	3.500	0.000	0.869	3.088

**Note:** Entrepreneurial intention toward information technology and telecommunications businesses is the dependent variable. Number of observations = 788, R-squared = 0.069, Adj R-squared = 0.060, Prob > F = 0.000.

**Fig 2:**
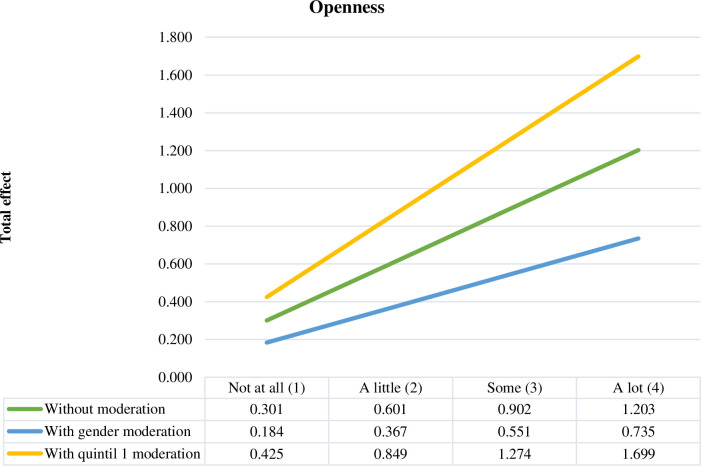
Total effect of openness.

Fig 2 shows the total effect of the personality trait of openness on the intention to undertake information technology and telecommunications businesses, representing the moderating effect of gender and family income level. Female gender also decreases the effect of openness on entrepreneurial intentions, and belonging to the first income quintile increases it.

Finally, Table 6 evaluates the moderating effect of female gender, the first income quartile and the centennial generation on the relationship between the conscientiousness personality trait and the intention to undertake information technology and telecommunications businesses. Again, the negative moderating effect of female gender (β = -0.119; P<0.01) and the positive moderating effect of lower family income (β = 0.117; P<0.01) are demonstrated. The moderating effect of belonging to the centennial generation is not supported (β = 0.063; P>0.10). Likewise, the regression model shows a good fit (Prob > F = 0.00).

**Table 6:** Regression model with moderation related to consciousness.

	Unstandardized coefficient β	Standardized coefficient β	Standard error	t	P>t	[95% Interval Conf.]	
**Agreeableness**	0.225	0.073	0.150	1.500	0.133	-0.069	0.519
**Neuroticism**	-0.241	-0.070	0.124	-1.940	0.053	-0.485	0.003
**Openness**	0.356	0.109	0.149	2.380	0.018	0.062	0.649
**Consciousness**	0.243	0.081	0.121	2.010	0.045	0.006	0.479
**Extraversion**	-0.093	-0.036	0.128	-0.720	0.470	-0.345	0.159
**Woman x Consciousness**	-0.119	-0.128	0.035	-3.420	0.001	-0.188	-0.051
**Quintile 1 x Consciousness**	0.117	0.117	0.037	3.190	0.001	0.045	0.188
**Centennial x Consciousness**	0.063	0.056	0.041	1.540	0.124	-0.017	0.143
**Constant**	1.944		0.565	3.440	0.001	0.835	3.054

**Note:** Entrepreneurial intention toward information technology and telecommunications businesses is the dependent variable. Number of observations = 788, R-squared = 0.071, Adj R-squared = 0.061, Prob > F = 0.000.

**Fig 3:**
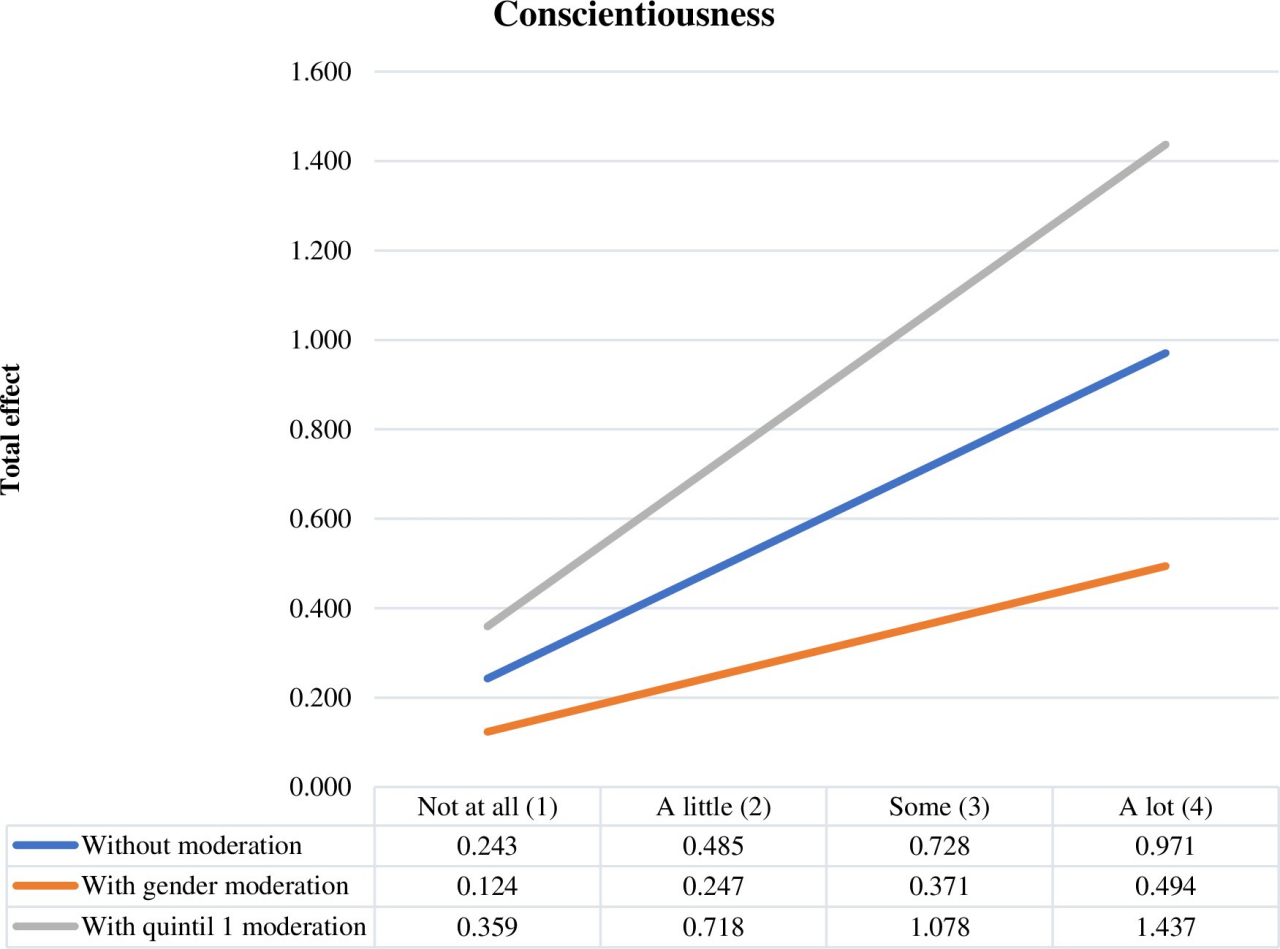
Total effect of consciousness.

Fig 3 represents the total effect of personality trait conscientiousness on the intention to undertake information technology and telecommunications businesses, showing the moderating effect of gender and income level. As shown in Figs 1 and 2, female gender reduces the effect of the conscientiousness trait on entrepreneurial intentions, and belonging to the first income quintile increases this effect.

## Discussion

The aim of this study was to evaluate the influence of the personality traits of university students in Latin America on their intention to undertake information technology and telecommunications ventures by analyzing the moderating effect of female gender, family income in the first quintile, and belonging to the centennial generation. The overall findings presented in Table 1 show that personality traits influence entrepreneurial intentions, suggesting that a unique set of psychological factors, mainly openness, conscientiousness and neuroticism, may be important for finding new opportunities and ways of organizing and developing information technology and telecommunications businesses in this region. The significant regression coefficients with 95% (P<0.05) confidence associated with neuroticism, openness and conscientiousness support this approach. According to these findings, Hypothesis 1 is verified. Moreover, the results of Tables 2, 3 and 4 support the moderating effect of female gender and lower family income with 99% (P<0.01) confidence in relation to Hypothesis 2 and Hypothesis 3 of this research. Finally, the evidence presented in Tables 2, 3 and 4 rejects the moderating effect of the centennial generation since the multiplicative coefficients linked to neuroticism, openness and conscientiousness are not significant (P>0.10); therefore, Hypothesis 4 is rejected.

The results are consistent with previous evidence about the effects of personality traits on entrepreneurial intention. Some studies have supported the positive influence of openness and conscientiousness traits [6,31,32]. In this sense, Presenza et al. [5] stated that tourism start-up entrepreneurs tend to be friendly, organized and unconventional; such characteristics are associated with the personality traits of extraversion, conscientiousness and openness in the Big Five model. In particular, the results for conscientiousness, which is related to self-confidence, efficiency and organization, are consistent with entrepreneurs’ tendency to be hardworking and persevering [81,82]. Moreover, Lent and Brown [83] stated that openness may be necessary for situations that require openness to different options; therefore, it is consistent that open-minded students express a higher intention to undertake information technology and telecommunications ventures.

An important difference in the results of this study compared to some previous research is that the positive influence of the extraversion trait, which is associated with positive emotions, sociability, cheerful, energetic and assertive behaviors, was not recognized. P values higher than 0.10 do not support the significance of regression coefficients. One interpretation of this finding is that more introverted people tend to perform functions in information technology because this business is related to programmers who, to a greater extent, tend to perform their work individually. In this regard, the tendency for software engineers to be introverted is supported [84]. The negative influence of neuroticism can be understood in terms of its association with negative emotions and personal fears [85], such as a greater fear of failure [86]. Additionally, the trait of neuroticism has been linked with a lack of self-confidence in the personal capability to effectively execute tasks, which represents low self-efficacy [87]. Both personal characteristics, fear of failure and low self-efficacy, have been supported as important determinants that inhibit entrepreneurial intention in previous research [88–91].

In addition, the differences by gender found in this study are consistent with previous findings that support lower entrepreneurial intention of women [92,93], mainly due to differences in gender roles in societies. In this regard, Tong [94] stated that women have weaker entrepreneurial intention than men due to factors such as education, life cycle, social gender roles and gender culture. Boubker et al. [95] argued that social norms have a negative effect on young women’s intention to start a business. Governments are called upon to propose follow-up programs for women entrepreneurs to improve their level of entrepreneurial intention. Furthermore, the negative moderation of gender evidenced in this study is in line with previous research that has supported a negative moderating effect of female gender or a positive moderating effect of male gender [44,96]. Finally, the positive moderation of lower family income shown in this study could be explained by the higher prevalence of need-driven entrepreneurship in Latin America [97], that is, a greater tendency toward entrepreneurship driven by the search for subsistence income [98]. In this sense, the findings of this research suggest that university students in Latin American countries who have lower family income appreciate the creation of information technology and telecommunications businesses as an alternative to reduce their poverty levels.

This study’s findings have several implications for academic institutions, university students and local government politicians in Latin America. These findings can guide the identification of students with higher and lower propensities to undertake this type of business. This is highly relevant for the social and technological development of the countries in the region since, after the COVID-19 pandemic, the digital transformation of companies accelerated, requiring suppliers of software and information and communication technologies that can be offered by new entrepreneurs with university education. Therefore, this research may orient the design and implementation of intervention programs to strengthen entrepreneurship adapted to the personality characteristics and demographics of Latin American university students. The results of this study show that the allocation of university support to information technology and telecommunications entrepreneurship can be evaluated considering students’ personality profiles, gender and family income level.

In particular, lecturers can evaluate students’ personality traits to understand their orientation toward creating information technology and telecommunications businesses. Lecturers could also give priority to students with higher development of the traits of openness and conscientiousness when guiding university projects associated with business creation. In addition, given that openness is the personality trait with the strongest positive effect on entrepreneurial intention, it is important that students are encouraged to positively value new experiences and the use of new technologies. Therefore, students should be motivated to become more involved in technological issues such as information technology and telecommunications since they must be open to accepting new perspectives to solve technological challenges. Moreover, women with high levels of conscientiousness and openness should receive special support in terms of training, funding, equipment and infrastructure to promote female entrepreneurship in information technology and telecommunications. Finally, colleges and universities can update their curricula to encourage students to undertake entrepreneurship in information technology and telecommunications businesses considering such gender differences.

## Conclusions

This research evaluates the influence of the personality traits of Latin American university students on their intention to start information technology and telecommunications businesses. It was determined that personality traits influence entrepreneurial intentions considering that a unique set of psychological factors, mainly related to openness, conscientiousness and neuroticism, can be important for finding new opportunities and ways to organize and develop information technology and telecommunications businesses in this region. At the same time, this research reveals how Latin American students with the greatest intention of starting information technology and telecommunications businesses show an original and complex combination of personality traits, which in turn is moderated by their gender and income level. Likewise, it can be concluded that university students from Latin American countries who have lower family income value the creation of information technology and telecommunications companies; this can be considered an opportunity since the creation of companies provides an alternative to reduce poverty levels. Moreover, the results of this study indicate that in entrepreneurship, gender differences influence entrepreneurial intention since lower entrepreneurial intention was found in women, which may be due to differences in gender roles in societies.

From a theoretical perspective, the results confirm a large part of the existing knowledge in the literature on entrepreneurship regarding the personality traits that affect the intention to undertake entrepreneurship. Therefore, it is possible to build education and training dynamics that tend toward culture entrepreneurship for students. In this new scenario, new academic spaces can be formulated in the university that favor and encourage the entrepreneurial spirit. From a management perspective, the results are highly relevant for university administrators and others involved in training future technology entrepreneurs since a better understanding of the main traits that affect entrepreneurial intention will contribute to the development of educational programs and interventions as well as more effective training. Therefore, if professors evaluate the personality traits of students and give priority to students with greater development of the traits of openness and awareness, they will be able to guide the formulation and execution of university projects associated with the creation of information technology and telecommunications companies.

## Limitations and future research

First, the sample of students was exclusively from Chile and Ecuador, and the sample size included 788 students, which can be considered a limitation. However, this number of responses is greater than the number of responses used in several recent studies that have evaluated students´ entrepreneurial intentions [99–102]. In addition, the sample analyzed included responses from students of different genders, income and age ranges, which improves the sample’s representativeness. Conducting similar research in other Latin American countries would improve generalization and reveal some national variations. Second, although this research highlighted the importance of information technology and telecommunications businesses, other business areas, such as tourism, construction, commerce and services, are highly important in Latin American economies. Therefore, the influence of personality on the intention to undertake these businesses could be studied. Third, the moderation of other important variables previously studied, such as participation in entrepreneurial education programs [103] and entrepreneurial self-efficacy [104], could be assessed in future studies. Fourth, this study applied a quantitative approach by highlighting the relevant relationships between the elements investigated. Although this is a useful approach, it does not provide a deeper understanding of the results. Therefore, future studies that combine quantitative and qualitative analyses could complement the presented findings.
